# Laparoscopic remnant cholecystectomy for calculi in the remnant gallbladder following subtotal-cholecystectomy: a report of two cases

**DOI:** 10.1186/s40792-021-01333-1

**Published:** 2021-11-29

**Authors:** Takeshi Utsunomiya, Katsunori Sakamoto, Kyousei Sogabe, Ryoichi Takenaka, Tatsuya Hayashi, Fumiya Ogura, Hisato Yamamoto, Naoki Ishida, Taro Nakamura, Akimasa Sakamoto, Miku Iwata, Chihiro Ito, Takashi Matsui, Yusuke Nishi, Mikiya Shine, Mio Uraoka, Tomoyuki Nagaoka, Kei Tamura, Naotake Funamizu, Kohei Ogawa, Yasutsugu Takada

**Affiliations:** 1 Department of Hepato-Biliary-Pancreatic and Breast Surgery, Ehime University Graduate School of Medicine, 454 Shitsukawa, Toon, Ehime 791-0295 Japan; 2 Department of Surgery, Uwajima City Hospital, 1-1 Gotenmachi, Ehime, 798-8510 Japan; 3 Department of Surgery, Seiyo Municipal Hospital, 147-1 Nagaosa, Uwa town, Seiyo, Ehime 797-0029 Japan

**Keywords:** Remnant gallbladder calculi, Fluorescence imaging, Laparoscopic operation

## Abstract

Two cases of laparoscopic remnant cholecystectomy using near-infrared fluorescence cholangiography (NIFC) for remnant gallbladder calculi following subtotal-cholecystectomy are reported. Case 1: a 36-year-old woman was referred to our hospital with acute abdomen. Computed tomography showed remnant gallbladder calculi, with detected no other findings as the cause of the abdominal pain. For intraoperative exploration of the biliary anatomy, 0.25 mg/kg of indocyanine green (ICG) was administered intravenously the day before the operation. NIFC clearly showed the common bile duct and enabled safe laparoscopic remnant cholecystectomy. She was free from symptoms after the operation. Case 2: a 40-year-old woman was referred to our hospital with epigastralgia due to remnant gallbladder calculi after open cholecystectomy. ICG was administered intravenously the day before the operation. Severe adhesions were observed in the upper abdominal cavity and there was tight adherence of the duodenum to the remnant gallbladder. NIFC showed a clear margin that appeared to be the margin between the duodenum and remnant gallbladder. However, dissection of the margin observed by NIFC caused perforation of the duodenum. The clear margin seen with NIFC was likely due to visualization of the gallbladder through the duodenum. Although NIFC is a useful modality for confirming the intraoperative biliary anatomy, it is important not to rely too heavily on NIFC alone, which may lead to misinterpretation of the anatomy.

## Background

The symptoms of post-cholecystectomy syndrome (PCS) are similar to those experienced prior to cholecystectomy [1]. Remnant gallbladder and remnant cystic duct calculi are rare causes of PCS [2–4]. The rate of remnant gallbladder calculi is much higher following subtotal-cholecystectomy (SC) than conventional cholecystectomy [5]. Although SC can cause remnant cholecystitis [6], it is often selected as the treatment option if cholecystectomy is difficult [7, 8]. The cases of two patients who underwent laparoscopic surgery with near-infrared fluorescence cholangiography (NIFC) for PCS caused by remnant gallbladder and cystic duct calculi after SC are reported.

## Case presentations

**Fig. 1:**
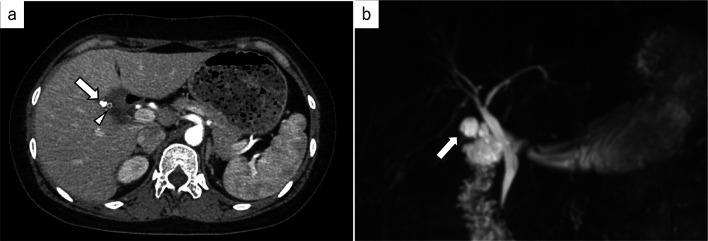
Abdominal contrast-enhanced CT and MRCP images. **a** Contrast-enhanced axial CT image: a calculus is seen in the remnant cystic duct (arrowhead). The staple line of the prior subtotal-cholecystectomy (arrow) is apparent on the fundal side of the remnant gallbladder. **b** MRCP image: no choledocholithiasis or intrahepatic bile duct dilation is observed. The arrow indicates the remnant gallbladder

**Fig. 2:**
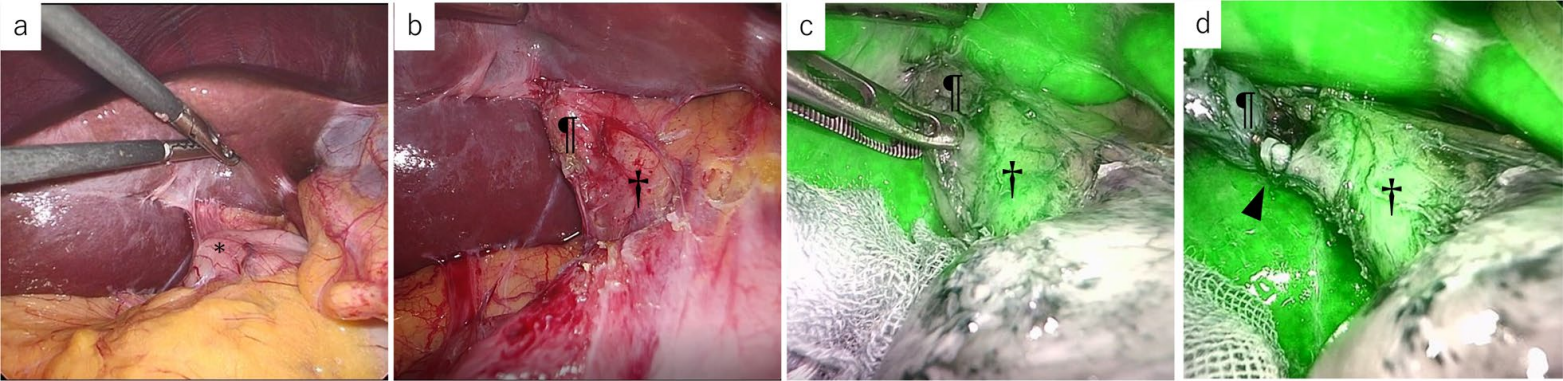
Intraoperative findings. **a** There is some adhesion of the duodenum (*) to the hepatoduodenal ligament. **b** Hilar region observed under white light. Each symbol corresponds to a structure in **c**. **c** The common bile duct is clearly visible (†). The remnant gallbladder shows weak fluorescence (¶). **d** The cystic duct (arrowhead) has been encircled and clipped. The cystic duct shows weak fluorescence

**Fig. 3:**
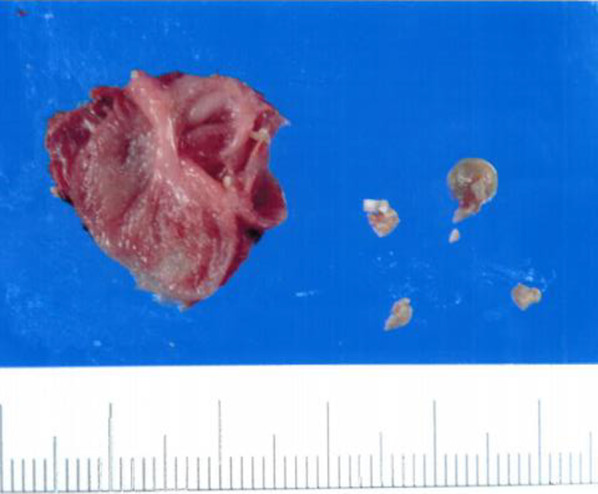
Resected specimen. There is thickening of the residual gallbladder wall due to chronic inflammation

*Case 1:* A 36-year-old woman was referred to our hospital with acute abdominal pain. She had undergone urgent laparoscopic cholecystectomy at our hospital 2 years previously for acute cholecystitis. Because of severe inflammation around Calot’s triangle, laparoscopic SC was performed at the previous operation. Her blood biochemical data at presentation to our hospital were as follows: white blood cells, 8430/µL (4000–9000/µL); hemoglobin, 12.7 g/dL (11.0–15.0 g/dL); platelets, 28.6 × 10^4^/µL (15.0 × 10^4^–45.0 × 10^4^/µL); albumin, 4.2 g/dL (3.4–4.8 g/dL); total bilirubin, 0.7 mg/dL (0.2–1.2 mg/dL); AST, 540 U/L (13–33 U/L); ALT, 242 U/L (6–27 U/L); γ-GTP, 341 IU/L (6–46 IU/L); C-reactive protein (CRP), 0.24 mg/dL (0.00–0.30 mg/dL) and prothrombin time international normalized ratio (PT-INR), 0.95 (0.80–1.30). Contrast-enhanced computed tomography (CT) and magnetic resonance cholangiopancreatography (MRCP) showed remnant gallbladder calculi, but no choledocholithiasis (Fig. 1). Laparoscopic remnant cholecystectomy for the remnant gallbladder calculi was planned. The patient was administered 0.25 mg/kg of indocyanine green (ICG, Daiichi Sankyo Co., Japan, Tokyo) intravenously 16 h before surgery for NIFC. A trocar for the laparoscope was inserted into the umbilicus by the open method, and three trocars were placed in the right upper quadrant. The laparoscopic system was VISERA ELITE II OLYMPUS CLV-S200-IR (OLYMPUS Co., Japan, Tokyo). There was adhesion of the duodenum to the hepatoduodenal ligament (Fig. 2a). After adhesionectomy of the duodenum (Fig. 2b), the common bile duct was identified by NIFC (Fig. 2c). The remnant gallbladder was identified as a defect in the fluorescent image. Since the remnant cystic duct showed inflammatory thickening, it was divided after one ligation and two clips (Fig. 2d). Since the cystic artery was cauterized by Sonosurg (OLYMPUS Co.) at the first operation, it could not be identified in this operation. Cholangiography was performed through a preoperatively placed ENBD-tube to confirm the presence of calculi, but the tip of the tube migrated to the duodenum. No further intraoperative treatment was performed because the calculi were confirmed in the resected specimen. The surgical duration was 114 min and the estimated intraoperative blood loss was 10 mL. The resected specimen of the remnant gallbladder was 2.0 cm in diameter and the calculi were 5 mm in diameter (Fig. 3). The patient’s postoperative course was uneventful, and the patient was discharged on postoperative day 4. The abdominal pain and elevated liver enzymes improved immediately after surgery.

**Fig. 4:**
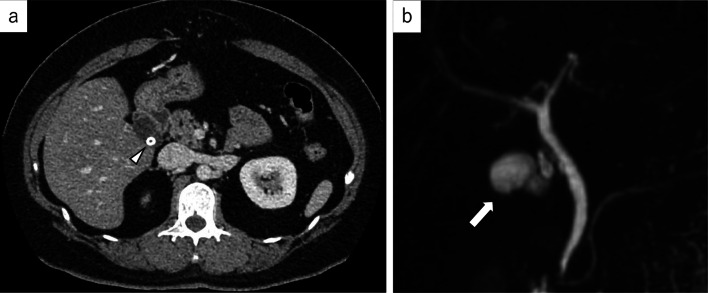
Contrast-enhanced CT of the abdomen and MRCP images. **a** Contrast-enhanced axial CT: a calculus is seen in the neck of the gallbladder (arrowhead). **b** MRCP: there are no common bile duct stones and no intrahepatic bile duct dilatation. The remnant gallbladder is indicated by the arrow

**Fig. 5:**
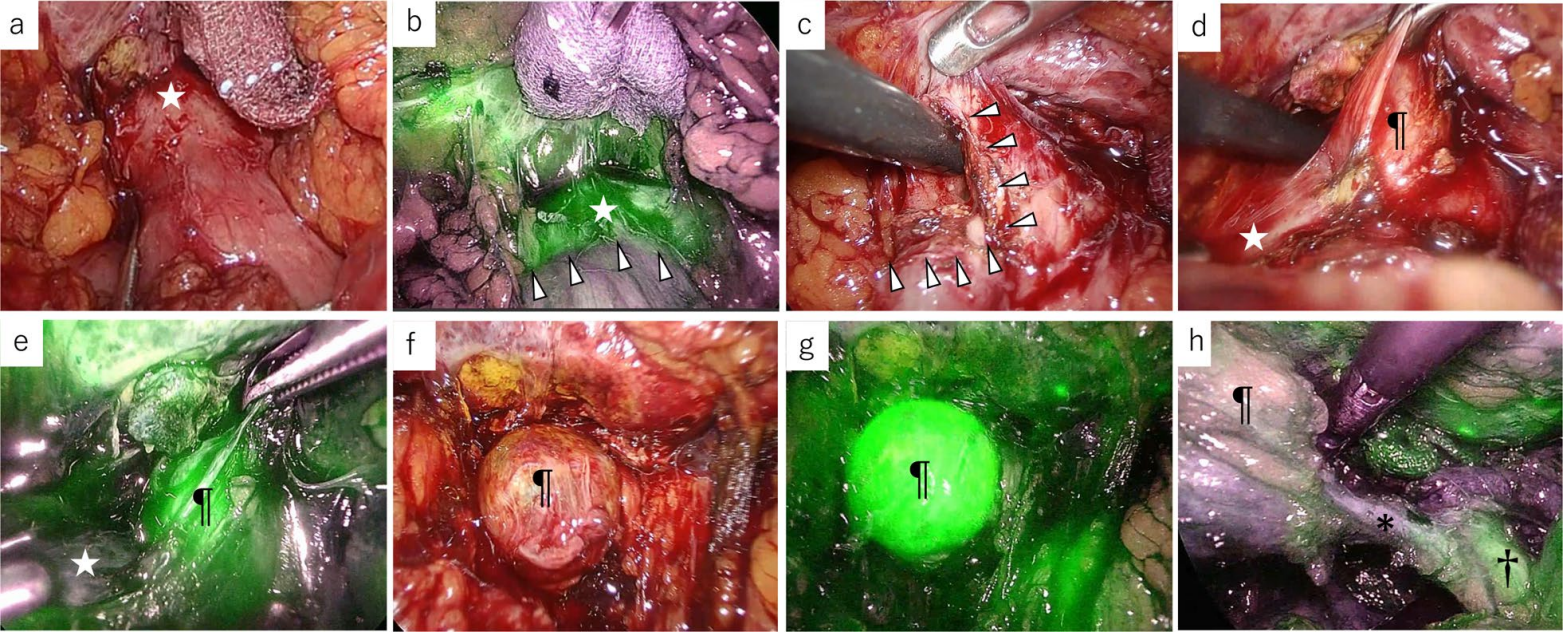
Intraoperative findings. **a** Laparoscopic image: there is adherence of the duodenum to the gallbladder bed. **b** NIFC image: NIFC shows a well-defined fluorescent border on the duodenum-like structure (arrowhead). **c** Location of the duodenal bulb perforation (arrowhead). A suction tube is inserted into the duodenum. **d** White light image: a part of the remnant gallbladder (¶). The white star indicates the duodenum. **e** Remnant gallbladder (¶) shows fluorescence. The white star indicates the duodenum. **f** White light image: the remnant gallbladder (¶) appears after adhesion dissection. **g** Remnant gallbladder (¶) shows high-intensity fluorescence after adhesion dissection. **h** The cystic duct (*) and common bile duct (†) are clearly recognizable by fluorescence. ¶, remnant gallbladder

**Fig. 6:**
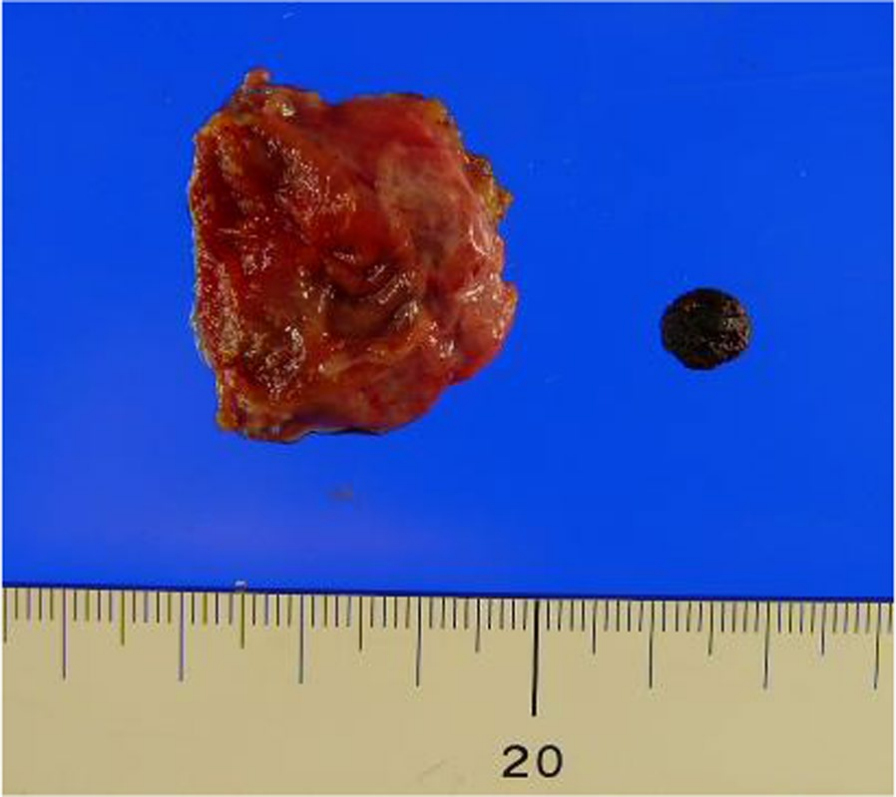
Resected specimen. There is thickening of the residual gallbladder wall. The diagnosis is chronic remnant cholecystitis

*Case 2:* A 40-year-old woman was referred to our hospital with epigastralgia caused by calculi in the remnant gallbladder. Ten years ago, she had undergone urgent open SC for severe acute cholecystitis with a midline incision. Her blood biochemical data at presentation to our hospital were as follows: white blood cells, 9600/µL (3500–9100/µL); hemoglobin: 14.7 g/dL (11.3–15.2 g/dL); platelets, 32.2 × 10^4^/µL (13.1 × 10^4^–36.9 × 10^4^/µL); albumin, 4.5 g/dL (3.9–4.9 g/dL); total bilirubin, 0.6 mg/dL (0.1–1.1 mg/dL); AST, 21 U/L (9–37 U/L); ALT, 34 U/L (3–49 U/L); γ-GTP, 53 IU/L (6–71 IU/L); CRP, 0.13 mg/dL (0.00–0.20 mg/dL) and PT-INR, 0.93 (0.89–1.07). Contrast-enhanced CT and MRCP showed the remnant gallbladder and a calculus, but revealed no other findings as the cause of the upper abdominal pain (Fig. 4). Laparoscopic remnant cholecystectomy was planned. ICG was administered intravenously, as in Case 1. Trocar placement and the laparoscopic system were the same as for Case 1. There was extensive adherence of the greater omentum from beneath the midline incision to the caudal surface of the liver. After dissecting the adhesions, a duodenum-like structure adherent to the gallbladder bed and the hepatoduodenal ligament was seen (Fig. 5a). NIFC clearly showed the border of the duodenum-like structure (Fig. 5b). Accordingly, the border was dissected as the margin of the duodenum and remnant gallbladder. However, dissection at the border caused injury to the bulb of the duodenum (Fig. 5c). The fluorescent part of the duodenum-like structure was in fact a part of the duodenum, because the fluorescence of the remnant gallbladder penetrated the duodenum. Thus, after adhesionectomy of the duodenum, the remnant gallbladder could be detected at the dorsal side of the duodenum (Fig. 5d, e). The cystic duct, cystic artery and common bile duct were identified (Fig. 5f), and the cystic artery and cystic duct were divided. Intraoperatively, the tip of a nasogastric tube was placed beyond the bulb of the duodenum into the descending duodenum for decompression of the suture site, and the duodenal wall was sutured primarily. Surgical duration was 280 min, and intraoperative estimated blood loss was 100 mL. The resected remnant gallbladder was about 3.0 cm in diameter, and the calculus was 6 mm in diameter (Fig. 6). The nasogastric tube was removed on the 3rd postoperative day, and the intraoperatively inserted drainage tube was removed on the 5th postoperative day. Oral intake was initiated from the 6th postoperative day. The patient was discharged on the 15th postoperative day with an uneventful course. The patient had no abdominal pain after the operation.

## Discussion

Remnant cystic duct/gallbladder calculi have been reported as a rare cause of PCS [2–4, 6]. The rate of remnant cystic duct/gallbladder calculi is reported to be 4.19% following SC [5], and the rate of remnant cholecystitis after SC is 1.4% [6]. In studies that have reported treatment of remnant cholecystitis [6] and symptomatic remnant calculi [8–12], the majority of patients required remnant cholecystectomy. Prior to remnant cholecystectomy, preoperative examination of the biliary anatomy is important to avoid bile duct injury. In addition, intraoperative identification of the biliary anatomy is also vital, especially in cases with severe adhesions. Some reports have suggested the usefulness of intraoperative cholangiography using an endoscopic nasobiliary drainage tube (ENBD-tube) to prevent bile duct injury [13, 14]. However, intraoperative cholangiography is a complex and time-consuming procedure, and ENBD-tube placement carries the risk of endoscope-induced pancreatitis. Although in the present Case 1, an ENBD-tube was placed before the operation, the tip of the tube migrated to the duodenum intraoperatively. NIFC has been reported as a useful modality for visualizing the extrahepatic biliary structures during laparoscopic surgery [15]. The amount and timing of ICG administration for intraoperative NIFC for detecting the biliary structures have been reported previously. A recent report has suggested that the present method is feasible for the visualization of biliary structures [16], as it was in the present two cases.

Intraoperative identification of the cystic duct is useful for safe laparoscopic cholecystectomy. In Case 1, however, the cystic duct calculi could not be detected radiologically, and the cystic duct could not be detected by endoscopic retrograde cholangiopancreatography (ERCP) before the operation. Similarly, the cystic duct was not detected by NIFC. However, a previous study suggested that the cystic duct can be well-visualized by NIFC [15]. In case 1, the remnant gallbladder and cystic duct could not be visualized by NIFC due to impacted stones. The important point is that the common bile duct could be identified as a well-contrasted structure and the operation was completed safely.

Adhesions around the hepatic hilum and duodenum were more severe in Case 2 than in Case 1 because the prior operation had been performed by the open approach due to severe inflammation. In fact, preoperative imaging had shown that the duodenum abutted the remnant gallbladder. The intention has been to identify the margin between the remnant gallbladder and the duodenum using NIFC; however, fluorescence of the remnant gallbladder was observed through the duodenal wall during the operation, and it was misinterpreted as the margin between the remnant gallbladder and the duodenum existing on the duodenum. Although the tissue penetration ability of near-infrared light is limited to 5–10 mm [17], thinning of the duodenum by compression of the remnant gallbladder and strong fluorescence of the remnant gallbladder might cause the appearance of a fluorescent remnant gallbladder through the duodenum. The degree of fluorescence of the biliary anatomy was different between Case 1 and Case 2. Factors affecting fluorescence include the distance between the fluorescent object and the camera, as well as the thickness of the tissue, which can be influenced by different laparoscopic imaging systems [18]. In inflammatory cases, such as the present cases, it may be difficult to achieve adequate tissue extension in postoperative scar tissue. Therefore, we should take into account individual differences in fluorescence intensity when evaluating anatomical structures by NIFC. Despite its problems, NIFC is useful in understanding intraoperative biliary anatomy, and Matsudaira et al. have reported its usefulness in laparoscopic surgery for remnant calculus [19]. The present report differs from that of Matsudaira et al. in that our experience of the pitfalls in laparoscopic surgery of the remnant gallbladder is reported. This, together with the previous report by Matsudaira et al., should be useful for the future use of NIFC.

Finally, the most important thing is the macroscopic findings seen by the surgeon's unassisted vision, and NIFC should be used just as a support. In case 2, after duodenal perforation, the remnant gallbladder was recognized behind the duodenum, and the operation was completed laparoscopically. However, at the same time, in cases with severe adhesions after previous open emergent cholecystectomy, if an accident occurred and the residual gallbladder could not be identified, tactile recognition by the open approach would help surgeons and be safer.

## Conclusion

NIFC is a useful modality for identifying the extrahepatic biliary tract during laparoscopic remnant cholecystectomy. However, when there are severe adhesions around the remnant gallbladder, it is important to carefully evaluate the fluorescent appearance prior to dissection of the remnant gallbladder.

## Data Availability

Not applicable.

## Abbreviations

PCS: Post-cholecystectomy syndrome
SC: Subtotal-cholecystectomy
CT: Computed tomography
MRCP: Magnetic resonance cholangiopancreatography
NIFC: Near-infrared fluorescent cholangiography
